# The role of magnesium oxide foliar sprays in enhancing mint (*Mentha crispa* L.) tolerance to cadmium stress

**DOI:** 10.1038/s41598-024-65853-0

**Published:** 2024-06-27

**Authors:** Soheil Khanchi, Seyed Hamed Hashemi Khabir, Seyed Hatef Hashemi Khabir, Reza Golmoghani Asl, Saeedeh Rahimzadeh

**Affiliations:** 1 Department of Agronomy, Islamic Azad University of Sanandaj, Sanandaj, Iran; 2 Department of Agronomy, Islamic Azad University of Khoy, Khoy, Iran; 3 Department of Agronomy and Plant Breeding, Islamic Azad University of Tabriz, Tabriz, Iran; 4 Department of Plant Eco-Physiology, Faculty of Agriculture, University of Tabriz, Tabriz, Iran

**Keywords:** Essential oil, Magnesium, Oxidative stress, Rhizosphere toxicity, Root activity, Plant sciences, Plant physiology, Plant stress responses

## Abstract

This study investigates using magnesium foliar spray to enhance mint plants' growth and physiological performance under cadmium toxicity. It examines the effects of foliar application of magnesium oxide (40 mg L^−1^), in both nano and bulk forms, on mint plants exposed to cadmium stress (60 mg kg^−1^ soil). Cadmium stress reduced root growth and activity, plant biomass (32%), leaf hydration (19%), chlorophyll levels (27%), magnesium content (51%), and essential oil yield (35%), while increasing oxidative and osmotic stress in leaf tissues. Foliar application of magnesium increased root growth (32%), plant biomass, essential oil production (17%), leaf area (24%), chlorophyll content (10%), soluble sugar synthesis (33%), and antioxidant enzyme activity, and reduced lipid peroxidation and osmotic stress. Although the nano form of magnesium enhanced magnesium absorption, its impact on growth and physiological performance was not significantly different from the bulk form. Therefore, foliar application of both forms improves plants' ability to withstand cadmium toxicity. However, the study is limited by its focus on a single plant species and specific environmental conditions, which may affect the generalizability of the results. The long-term sustainability of such treatments could provide a more comprehensive understanding of magnesium's role in mitigating heavy metal stress in plants.

## Introduction

Industrialization has accelerated the production of compounds containing heavy metals across various sectors over time^1^. The increasing presence of heavy metals in soil has adverse effects on plant growth and development, which poses a significant challenge for sustainable agriculture^2^. Among these metal contaminants, cadmium (Cd), a potentially toxic element, has increased in cultivated soils due to the excessive use of chemical fertilizers, particularly phosphate fertilizers, and pesticides^3^. Cd contamination presents substantial threats to soil quality and human health, especially in edible agricultural produce^4,5^. Given its high solubility and assimilation, Cd can easily enter plants through the roots, thereby hindering plant growth and productivity^6^. Cadmium disrupts various metabolic processes, including photosynthesis, respiration, and mineral nutrient uptake. This ultimately leads to reduced growth, yield, and overall plant health^6^. A thorough understanding of the mechanisms behind these disruptions can help in developing strategies to mitigate the negative impacts of cadmium stress on plant productivity.

The extent of plants' tolerance to acute Cd toxicity appears to be closely associated with their antioxidant capacity^7^. Furthermore, there are clear associations between exposure to Cd stress in the environment, antioxidant capacity, and redox equilibrium in plants. The production of Reactive Oxygen Species (ROS) leads to non-targeted oxidative damage to proteins, lipids, nucleic acids, and other biological components^8^. Wan and Zhang^9^ and Loix et al.^10^ suggest that the stiffening of the cell wall and the decrease in cell elongation in plants exposed to high levels of Cd are likely caused by damage to the cell wall components. A recent study by Farhangi-Abriz and Ghassemi-Golezani^11^ demonstrated that, in addition to mitigating oxidative stress, enhancing plant osmotic adjustment plays a crucial role in resistance to cadmium stress. Improved nutritional conditions enable plants to thrive and function more effectively under cadmium stress.

Magnesium (Mg) is a crucial nutrient for the growth and development of plants. It is involved in various cellular processes, such as chlorophyll production, photosynthesis, and the synthesis of carbohydrates and proteins. It also serves as a cofactor for multiple enzymes in plant metabolism^12^. Magnesium effectively mitigates physiological and biochemical stress in plants exposed to heavy metal toxicity^13,14^. Given their chemical resemblance and shared transporters with heavy metals, nutrients like magnesium play a significant role in enhancing tolerance to heavy metal stress^15^. Nanomaterials, such as metallic nanoparticles (NPs), offer a promising avenue for stress alleviation^16^. Nanoparticles possessing at least one dimension below 100 nm exhibit numerous advantages, including a large specific surface area, numerous surface reaction sites, efficient catalytic capabilities, and unique optical and magnetic properties^17^. The intelligent synthesis of nanomaterials aids in minimizing nutrient losses. These nanomaterials consist of both macro and micronutrients, enabling efficient nutrient delivery to plants and providing a potential remedy for mitigating the detrimental impacts of traditional chemical fertilizers^18^.

The foliar application of nutrients represents a promising strategy for addressing nutrient deficiencies and mitigating the adverse impacts of abiotic stresses^19^. Applying nutrients through foliar spraying facilitates their swift translocation, reducing plant physiological stress by delivering them directly to the point of assimilation^20^. When nutrients in the form of NPs are sprayed onto leaves, they enter through stomata and are subsequently transported to various plant components via apoplastic and symplastic pathways. Numerous investigations have demonstrated that foliar application of NPs contributes to enhanced plant photosynthesis, increased activity of antioxidant enzymes, and decreased accumulation of reactive oxygen species, thereby alleviating stress caused by heavy metals^17,21,22^. In a study by Kanjana^23^, it was found that the foliar application of MgO nanoparticles resulted in increased concentrations of macronutrients (N, P, K) and Mg in cotton plants. Furthermore, Faizan et al.^24^ reported that the application of magnesium oxide nanoparticles via foliar spraying alleviated arsenic toxicity in soybean plants, resulting in a 15% increase in plant biomass.

Mint, a medicinal plant with numerous potential health benefits, is used in both the food and pharmaceutical industries. Its invigorating flavor and aroma have made it a sought-after option for enhancing a wide range of products, including confectionery, gum, and beverages. Beyond its culinary appeal, mint is recognized for its effectiveness in alleviating stomach discomfort and chest pain. Notably, the essential oil extracted from mint plants shows remarkable potential to improve digestion, reduce inflammation, and relieve allergic symptoms^25,26^. Cultivating medicinal plants for essential oil extraction is a highly effective strategy for enhancing plant production in the presence of heavy metal stress. An important characteristic of medicinal plants like mint is that heavy metals, including cadmium, do not penetrate their essential oils. This property makes mint particularly advantageous for cultivation in contaminated soils. Cultivating mint in cadmium-polluted soils without compromising the quality of its essential oil supports sustainable agricultural practices. This can help farmers use contaminated lands without the risk of producing unsafe or low-quality products. However, the presence of these contaminants may still hinder the growth and physiological efficiency of medicinal plants. Among the essential nutrients for plants, magnesium emerges as a powerful contributor to improving growth and productivity, especially in the presence of Cd toxicity. There is still a lack of studies investigating the impact of magnesium on enhancing plants' resistance to environmental stresses, such as cadmium toxicity. Against this backdrop, this research pioneers to find out whether foliar application of magnesium (both bulk and nano forms) improves the growth, physiological performance, and essential oil yield of mint plants under cadmium stress. Moreover, is there a significant difference in the efficacy of bulk versus nano forms of magnesium in mitigating cadmium toxicity in mint plants? The findings of this research could be applied to develop sustainable agricultural practices by promoting the use of magnesium foliar sprays to alleviate heavy metal stress in crops. By selecting mint as the study plant, we aim to address a significant gap in understanding how to mitigate heavy metal stress in a crop of both medicinal and economic importance. This can help farmers utilize contaminated soils more effectively, maintaining crop yields and quality without requiring extensive soil remediation efforts.

## Materials and methods

### Experimental conditions

A factorial arrangement (3 × 2) was implemented within a randomized complete block design, consisting of three replications, in a greenhouse setting. The greenhouse maintained average temperatures of 25/19 °C (day/night), air humidity at 39%, light intensity of 138 W m^−2^, and the daylight photoperiod was approximately 13 h. Each pot (25 × 25 cm) contained soil that had initially been contaminated with cadmium, with concentrations of 0 and 60 mg CdSO_4_ kg^−1^ soil representing non-toxic and toxic conditions, respectively. After mixing soil with CdSO₄, the moisture content of the soil was adjusted to field capacity by adding water while continuously mixed to ensure even distribution. The soil was then incubated for 14 days. During this period, the soil moisture was maintained at field capacity. The potting soil was regularly mixed every 2–3 days to maintain uniform contamination and prevent anaerobic conditions. The key characteristics of the soil are outlined in Table 1. The cadmium concentration in the soil was selected based on a study conducted by Ghassemi-Golezani and Farhangi-Abriz^27^.

**Table 1:** Some physicochemical properties of the experimental soil.

Soil texture	pH	EC (dS m^−1^)	CEC (cmol kg^−1^)	OM	Total N	P	K	Mg	Mn	Zn	Fe	Cd-b	Cd-a
				(%)		(mg kg^−1^)							
Silty loam	7.2	2.11	18.3	1.5	0.06	47.2	131	19.6	9.6	0.58	2.8	0.11	29.6

*EC* electrical conductivity, *CEC* cation exchange capacity, *OM* organic mature, *Cd-b* cadmium content before contamination, *Cd-a* cadmium content after contamination.

Seeds of mint were sourced from Pakan Bazr company (Isfahan, Iran) and sown in all pots after being treated with fungicide. Proper soil moisture was maintained during the germination period. After sowing, tap water was added to the pots to achieve 100% field capacity (FC), with a 20% interval included to account for potential water loss. Yellow sticky traps were used to monitor the flying insect population. Upon the establishment of seedlings, the number of plants per pot was reduced to six to decrease competition and ensure proper growth. At this stage, a starter dose of a balanced fertilizer (20–20-20) with equal proportions of nitrogen (6.22% Nitrate; 3.88% Ammoniacal nitrogen; 9.90% Urea), phosphorus (20% P_2_O_5_), and potassium (K_2_O) (3 g L^−1^) was introduced to maintain soil fertility. Each pot received 300 ml of the fertilizer solution.

During the growth phase, 30 days after sowing (DAS), foliar applications were conducted using water, magnesium oxide nanoparticles (MgO-NPs), and bulk magnesium oxide particles (MgO-B), each at a concentration of 40 mg L^−1^. These treatments corresponded to the control (water), MgO nanoparticles, and bulk MgO particles, respectively. Hence, magnesium oxide (MgO) was applied in two distinct forms: MgO nanoparticles and bulk MgO particles. The All chemicals were procured from Nanoshel Company, a reputable supplier, located in Punjab, India. The nanoparticles had a purity of 99.9% and an average particle size (APS) of 0.04 µm (40 nm). In comparison, the bulk form of MgO had a purity level of 92.54% and an average particle size of 60 µm.

The dosages for foliar applications were established through preliminary testing. The foliar spray was applied using a hand-held sprayer with a flow rate of 1.5 L per minute. The spray nozzle produced droplets with an average size of 100 µm, ensuring uniform coverage of the leaves. The application was conducted under controlled environmental conditions, with air temperatures ranging from 22 to 25 °C and relative humidity between 60 and 70%. Only one foliar spray was conducted to evaluate the initial response of the plants to magnesium supplementation under cadmium stress. This approach allowed us to assess the immediate effects of foliar-applied magnesium without the confounding factors of multiple applications.

Upon reaching the flowering stage, one plant from each pot was harvested and subjected to oven-drying at 70 °C for 48 h for analysis of Cd and Mg. Fresh leaf samples from another plant were rapidly frozen in liquid nitrogen for the determination of physiological parameters. Leaf samples were collected from all parts of the plant. Given that sampling took place during the flowering stage, approximately 45 days after planting, all the leaves were in their youthful stage. Two additional plants were used to measure leaf area, CCI (chlorophyll content index), and LWC (leaf water content). Subsequently, the two remaining plants were harvested to assess biomass and extract essential oil from the vegetative components.

### Measurement of Cd and Mg in soil

Soil samples air-dried at room temperature and sieved through a 2 mm mesh to remove debris and larger particles. For Cd extraction, 10 g of soil were mixed with 50 mL of 0.01 M EDTA (Ethylenediaminetetraacetic acid) solution, and the mixture was shaken for 1 h. After shaking, the mixture was filtered through Whatman No. 42 filter paper, and the Cd concentration was determined using an Atomic Absorption Spectrophotometer at 228 nm. For Mg extraction, 10 g of soil was mixed with 50 mL of 1 M ammonium acetate (NH₄OAc, pH 7.0) and shaken for 30 min. The mixture was then filtered and the Mg concentration in the filtrate was measured at 285 nm^28^. The concentrations of cadmium (both before and after pollution), and magnesium are provided in Table 1.

### Root morphology and activity

The growth of roots was observed using GiA Roots software (Benfey laboratory, Duke University, Durham, North Carolina, USA) to analyze digitally captured root images. This software helps in studying the dynamics of root development and its responses to different environmental conditions or treatments. The images were all captured using a Sony Cyber-Shot digital camera (Sony, Japan)^29^. The images were imported into GiA Roots software that provides advanced algorithms to analyze root architecture and morphology. Pre-processing steps, such as image thresholding and noise reduction, were applied to enhance image quality and improve the accuracy of root detection. The root weight was determined by drying at 80 °C for 48 h, and root density in mg cm^−3^. For measuring root activity, approximately 700 mg of freshly harvested mint roots are combined with a phosphate buffer solution and 15 mL of a 0.4% triphenyl tetrazolium chloride (TTC) solution. Following thorough mixing, the mixture is incubated in the dark at room temperature for 3 h to allow for the reduction of TTC by the metabolic activity of the roots. Subsequently, to terminate the reaction, 3 mL of 1M sulfuric acid (H_2_SO_4_) is added. The resulting formazan product, indicative of metabolic activity, is quantified by measuring the absorbance of the samples at 485 nm using a spectrophotometer. This absorbance measurement enables the assessment of root activity, expressed as micrograms of TTC reduction per unit of time, providing valuable insights into the metabolic vitality of the mint roots under investigation^27^.

### Leaf area and chlorophyll content index

Leaf area (LA) was measured in cm^2^ per plant using a portable area meter (model ADCAM 300, United Kingdom). The chlorophyll content index (CCI) was determined using a chlorophyll meter (Opti-Sciences, CCM-200), which is designed to measure chlorophyll levels in plant leaves based on light absorbance properties. To conduct the measurement, a leaf from the mint plants was selected, and the chlorophyll meter was placed on the leaf surface, ensuring proper contact and alignment. The measurement was repeated three times on the same leaf to ensure accuracy, and the mean value was calculated as the CCI for that leaf. This process provided a representative measure of chlorophyll concentration in the leaf tissue.

### Leaf water content

The leaves of one mint plant were separated and weighed (FW: Fresh weight). After oven-drying at 80 °C for 48 h, leaf samples were reweighed (DW: Dry Weight). The leaf water content (LWC) was calculated using the below formula:

$$ {\text{LWC}} = \left( {{\text{FW }} - {\text{ DW }}/{\text{ FW}}} \right) \times {1}00 $$

### Antioxidant enzyme activities

For the measurement of antioxidant enzyme activities, frozen leaf samples weighing 0.5 g each were homogenized in a potassium phosphate buffer (50 mM, pH 7.0). This buffer solution, prepared by dissolving potassium phosphate salts in deionized water and adjusting the pH using a pH meter, contained 1% polyvinylpyrrolidone. The addition of polyvinylpyrrolidone helped prevent enzyme denaturation and proteolysis during the homogenization process. The resulting homogenate was then centrifuged to remove cell debris, and the supernatant was collected for the assessment of antioxidant enzyme activities. This included the determination of enzymatic activities such as catalase, peroxidase, and superoxide dismutase, providing insights into the plant's defense mechanisms against oxidative stress. For the estimation of peroxidase (POX) activity, the enzyme extract (0.1 mL) was mixed with a reaction mixture containing guaiacol (10 mM), potassium phosphate buffer (50 mM, pH 7.0), and H_2_O_2_ (30 mM). The change in absorbance was recorded at 470 nm for 2 min, with measurements taken every 20 s. The reaction mixture for catalase (CAT) activity contained reaction buffer (potassium phosphate buffer, 50 mM, pH 7.0), substrate (H_2_O_2_, 30 Mm), and 0.1 mL of the extracted enzyme. The CAT activity was measured by monitoring the changes in absorbance at 240 nm^30^. The activity of superoxide dismutase (SOD) was estimated by the enzyme's ability to inhibit the photochemical reduction of nitroblue tetrazolium. The reaction mixture contained reaction buffer (phosphate buffer, 50 mM, pH 7.8), riboflavin (2 µM), methionine (13 mM), ethylenediaminetetraacetic acid (EDTA, 0.1 mM), and nitroblue tetrazolium (75 µM) that reacts with superoxide anions as substrate. The absorbance was determined spectrophotometrically at 560 nm^31^.

### Lipid peroxidation rate

Fresh leaf tissues (100 mg) were homogenized in 2 mL of 0.1% trichloroacetic acid (TCA) and then centrifuged for 10 min at 12,000*g*. Next, 0.5 mL of the supernatant was mixed with 2 mL of 0.5% thiobarbituric acid (TBA) solution, prepared by dissolving 0.5 g of TBA in 100 mL of distilled water. The mixture was incubated in a water bath at 100 °C for 10 min to allow the reaction to occur. The reaction was then stopped using an ice bath. The sample's absorbance was recorded at 532 nm to measure the amount of malondialdehyde (MDA)-TBA complex formed, indicating lipid peroxidation levels. Measurements were corrected for non-specific turbidity by subtracting the absorbance at 600 nm^32^.

### ROS generation

Hydrogen peroxide (H_2_O_2_) content was determined using the method described by Velikova et al.^33^. Firstly, 0.1 g of fresh leaf tissue was homogenized with 2 mL of 0.1% (w/v) trichloroacetic acid at 4 °C. The homogenate was centrifuged at 12,000 g for 10 min. Then, the supernatant (0.5 ml) was thoroughly mixed with potassium phosphate buffer (0.5 ml) and potassium iodide 1 M (1 ml). The absorbance was recorded at 390 nm. For evaluating the O_2_^•−^ generation, 0.3 g of a fresh leaf was homogenized in a phosphate buffer solution. The homogenate was centrifuged for 20 min at 12,000*g*. Then, the supernatant was mixed with 0.9 ml of 65 mM PBS (phosphate-buffered saline) (pH 7.8) and 0.1 ml of 10.0 mM hydroxylamine hydrochloride. Then sulfanilamide and α-naphthylamine were added to the mixture. The absorbance was recorded at 530 nm^34^.

### Osmolytes

According to Bates et al.^35^, the proline content of leaves was assayed by reacting proline with ninhydrin. Leaf samples (50 mg FW) were homogenized in sulfosalicylic acid. Then, an equal volume of ninhydrin and glacial acetic acid solutions was added. The mixture was heated for 1 h at 100 °C, and then 4 mL of toluene was added. Finally, the absorbance was measured at 520 nm using a spectrophotometer. The soluble carbohydrates in mint leaves were assayed using the phenol–sulfuric acid method, as described by Dubois et al.^36^. Fresh leaf tissues were homogenized using ethanol. The filtered samples were mixed with phenol and sulfuric acid. The absorbance was measured at 485 nm using a UV–visible spectrophotometer.

### Cadmium and magnesium contents

Leaf and root samples were oven-dried at 80 °C for 48 h, ground, and then placed in a muffle furnace at 550 °C for 5 h. The ash samples were digested using 1 M nitric acid. The concentrations of Cd (in both roots and leaves) and Mg (only in leaves) were determined using an atomic absorption spectrophotometer (Shimadzu model: AA 7000, Kyoto, Japan)^37^.

### Essential oil content

After air-drying, the vegetative parts of the plant (stems and leaves) were hydro-distilled for 3 h using a Clevenger apparatus. The essential oils were then collected using anhydrous sodium sulfate. The yield of essential oil (mg plant^−1^) was reported based on the weight of oven-dried vegetative parts of plants (at 80 °C for 48 h) and their essential oil content.

### Statistical analysis

The SAS 9.1.3 software was utilized for comprehensive statistical analysis. Normality tests, including the Anderson–Darling test, were conducted to evaluate the normal distribution of the data. Furthermore, variance analysis was performed to assess the variability within the dataset. To deepen the analysis and understand the underlying mechanisms, a Principal Component Analysis (PCA) was conducted. PCA is a multivariate analysis method that allows for the exploration of complex relationships and patterns within the dataset, providing insights into the interactions between cadmium toxicity and magnesium application. The factorial arrangement, a key component of the analysis, involved the simultaneous study of two factors: Cd toxicity and Mg application. This facilitated the examination of their individual and interactive effects on plant physiology. Mean values were compared using the Tukey HSD (Honestly Significant Difference) test, which was chosen for its ability to control the family-wise error rate during multiple comparisons. Significance was determined at the *p* ≤ 0.05 level. The results were presented as mean ± standard error, providing a comprehensive overview of the experimental outcomes.

### Statement of compliance

This experimental research on plants complies with relevant institutional, national, and international guidelines and legislation.

## Results

### Root morphology and activity

**Figure 1:**
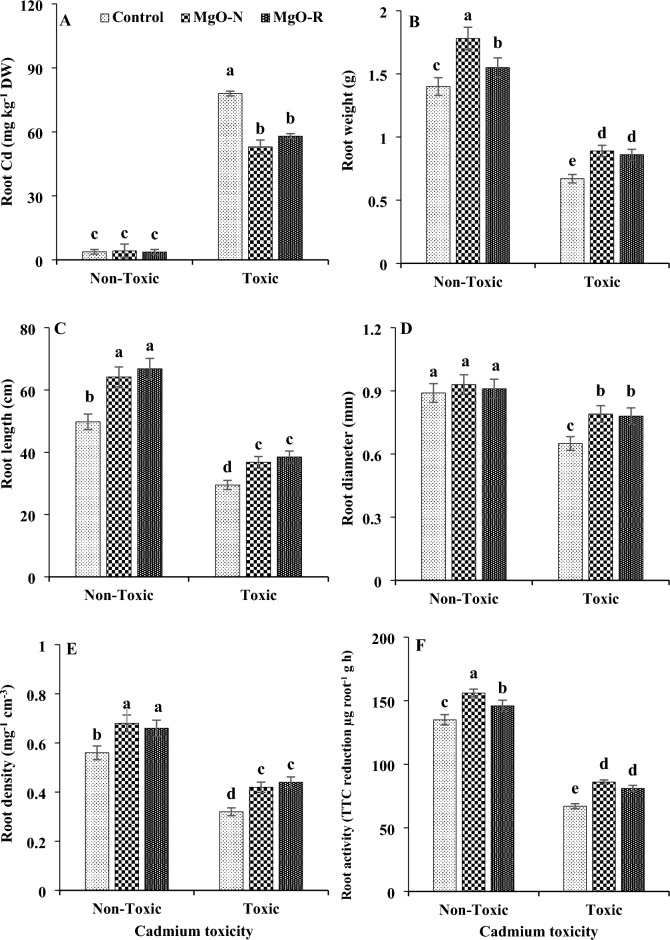
Changes in cadmium content (**A**), weight (**B**), length (**C**), diameter (**D**), density (**E**) and activity (F) of mint root under cadmium toxicity and foliar application of MgO particles. Data represent the average of three replicates (n = 3) ± standard error. Different letters indicate significant differences by Tukey HSD test at *p* ≤ 0.05.

Cadmium toxicity in the rhizosphere increased the concentration of this heavy metal in the root tissues of mint (Fig. 1). This stress decreased the weight, length, diameter, density, and root activity. Foliar spraying of plants with magnesium decreased the concentration of cadmium in the roots. Additionally, it increased root growth and activity. Magnesium foliar application improved mint root growth under both cadmium-toxic and non-toxic soil conditions. In most cases, both nano and bulk forms of magnesium had the same effects on decreasing the cadmium accumulation and increasing the root growth and activity of mint plants under Cd toxicity.

### Plant performance

**Figure 2:**
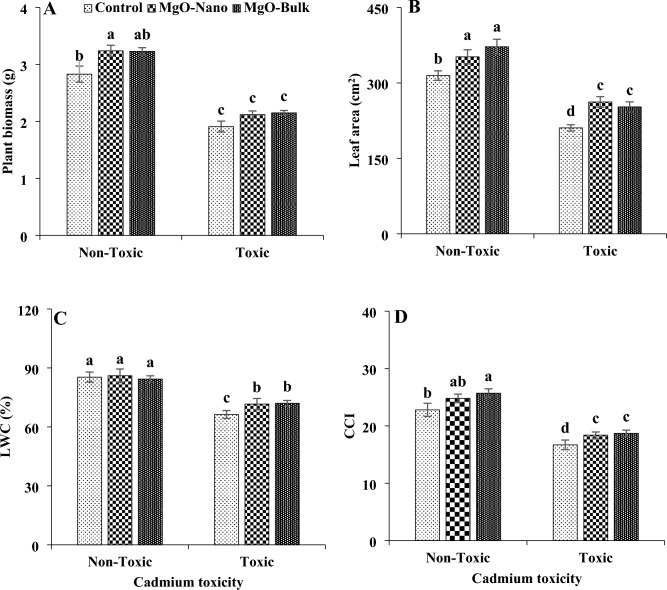
Changes in biomass (**A**), leaf area (**B**), LWC (**C**), and CCI (**D**) of mint plants under cadmium toxicity and foliar application of MgO particles. Data represent the average of three replicates (n = 3) ± standard error. Different letters indicate significant differences by Tukey HSD test at *p* ≤ 0.05. *LWC* leaf water content, *CCI* chlorophyll content index.

Cadmium toxicity resulted in a significant reduction in plant biomass, about 32%. It also decreased key parameters such as leaf area, chlorophyll content index, and leaf water content by 33, 26, and 19%, respectively (Fig. 2). The application of magnesium, whether in nano or bulk forms, exhibited a positive impact on plant biomass, LA, and CCI across both toxic and non-toxic conditions. Magnesium treatments had no significant effect on leaf water content under non-toxic conditions but led to an increment in leaf water content under cadmium toxicity. In the majority of cases, no significant distinction was observed between the effects of nano and bulk forms of magnesium on enhancing plant performance. Specifically, the application of nano magnesium under cadmium toxicity increased plant biomass by 11%, LA by 24%, CCI by 10%, and LWC by 5.3% compared to control plants. Under non-toxic conditions, magnesium oxide nanoparticles caused an increase in plant biomass (about 15%), LA (12%), and CCI (8%).

### Oxidative stress

**Figure 3:**
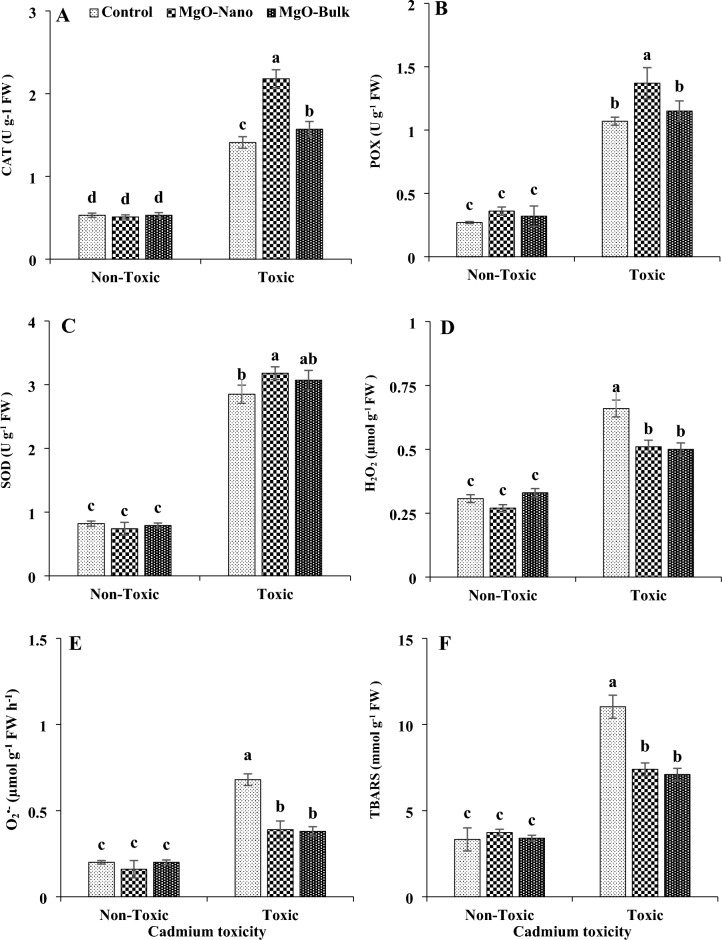
Changes in CAT (**A**), POX (**B**), SOD (**C**) activities and H_2_O_2_ (**D**), O_2_^•−^ (**E**), and TBARS (**F**) contents of mint leaves under cadmium toxicity and foliar application of MgO particles. Data represent the average of three replicates (n = 3) ± standard error. Different letters indicate significant differences by Tukey HSD test at *p* ≤ 0.05. *FW* fresh weight, *CAT* catalase, *POX* peroxidase, *SOD* superoxide dismutase, *TBARS* thiobarbituric acid reactive substances.

Cadmium toxicity led to notable elevations in the activities of catalase by approximately 1.6-fold, peroxidase by 2.9-fold, and superoxide dismutase by 2.4-fold. The increase in hydrogen peroxide, superoxide anion, and thiobarbituric acid reactive substances in plants exposed to cadmium toxicity was approximately 1.1-fold, 96%, and 42%, respectively (Fig. 3). The application of magnesium in both nano and bulk forms had no significant effect on antioxidant enzyme activity, ROS generation, or lipid peroxidation under non-toxic conditions. In contrast, the magnesium treatments demonstrated the ability to enhance antioxidant enzyme activity, reduce ROS production, and alleviate lipid peroxidation in mint plants under toxic conditions. Notably, the nano form of magnesium resulted in the most significant enhancement in the activities of SOD, CAT, and POX under cadmium toxicity. However, both the bulk and nano forms of magnesium demonstrated equivalent efficacy in reducing ROS generation and lipid peroxidation in mint leaves exposed to toxic conditions. When magnesium oxide nanoparticles were applied through foliar spraying, the activities of CAT, POX, and SOD increased by approximately 54%, 28%, and 11% respectively, under cadmium toxicity. Moreover, the application of magnesium oxide nanoparticles resulted in reductions of about 22% in H_2_O_2_ content, 42% in O_2_^·−^ content, and 32% in TBARS content within the leaves, compared to the control plants.

### Osmolytes

**Figure 4:**
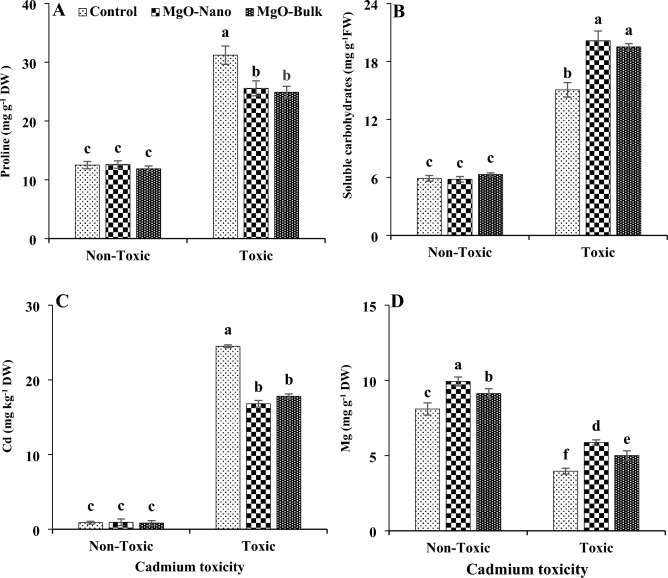
Changes in proline (**A**), soluble carbohydrates (**B**), Cd (**C**), and Mg (**D**) contents of mint leaves under cadmium toxicity and foliar application of MgO particles. Data represent the average of three replicates (n = 3) ± standard error. Different letters indicate significant differences by Tukey HSD test at *p* ≤ 0.05. *FW* fresh weight, *DW* dry weight, *Cd* cadmium, *Mg* magnesium.

Under cadmium toxicity, the proline content increased significantly (by about 1.5-fold), accompanied by a notable increase in soluble carbohydrates (by 1.5-fold) within leaf tissues (Fig. 4). When magnesium was applied in both nano and bulk forms, it resulted in a decrease in proline content while enhancing the levels of soluble carbohydrates in mint leaves subjected to cadmium toxicity. Nevertheless, magnesium treatments did not yield a significant impact on osmolyte production under non-toxic conditions. Both nano and bulk forms of magnesium demonstrated a similar effect on osmolyte content under cadmium toxicity. Furthermore, the application of magnesium, whether in nano or bulk forms, resulted in an approximate 18% decrease in proline content and a 33% increase in soluble carbohydrate content within leaf tissues under cadmium toxicity, compared to control plants.

### Cadmium and magnesium contents in plant leaves

The addition of cadmium into the soil resulted in an elevated cadmium concentration in leaf tissues, leading to a significant decrease in magnesium content by about 51% (Fig. 4). Applying both bulk and nano forms of magnesium oxide showed a reduction in the absorption of cadmium by plants exposed to cadmium toxicity. Foliar application facilitated an increase in the concentration of magnesium in plant tissues. Importantly, no statistically significant distinction was observed between the efficacy of bulk and nano forms of magnesium oxide particles in reducing cadmium accumulation in plant leaf tissues. In response to the application of magnesium oxide particles in nano and bulk forms, the cadmium content within leaf tissues experienced reductions of about 31% and 27%, respectively, compared to control plants. The foliar application of nano-sized magnesium oxide particles emerged as a more effective treatment for increasing magnesium content in leaves, both under normal and stressful conditions. Magnesium oxide nanoparticles increased the magnesium content of leaves by about 22% and 48% under non-toxic and toxic conditions, respectively, compared to control plants.

### Essential oil percentage and yield

**Figure 5:**
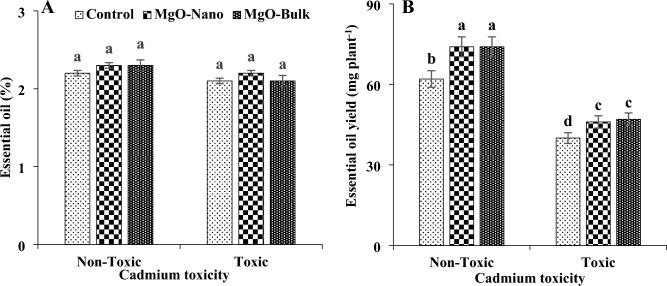
Changes in essential oil percentage (**A**) and yield (**B**) of mint plants under cadmium toxicity and foliar application of MgO particles. Data represent the average of three replicates (n = 3) ± standard error. Different letters indicate significant differences by Tukey HSD test at *p* ≤ 0.05.

Cadmium toxicity and foliar application of magnesium particles did not affect the percentage of essential oil in mint plants (Fig. 5). However, cadmium toxicity reduced the yield of essential oil in plants ranging from about 35% to 38%. The foliar application of magnesium oxide particles increased the essential oil yield of mint plants, both under normal and stressful conditions. The application of magnesium oxide particles led to an increase in essential oil production under normal and stressful conditions by about 19% and 17%. It is important to highlight that no statistically significant difference was observed between the effects of bulk and nano forms of magnesium oxide particles in terms of enhancing the yield of essential oil of mint plants.

### Principal component analysis

**Figure 6:**
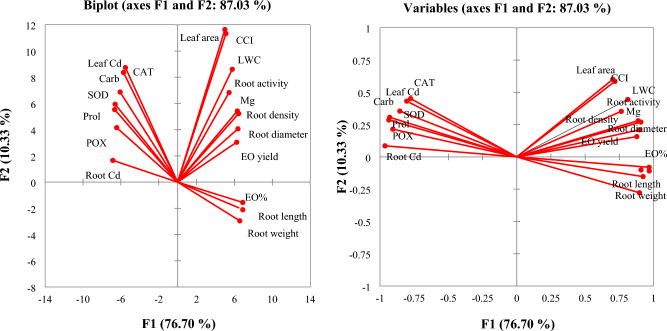
Principal component analysis (PCA) of the leaf and root parameters in mint plants. *EO* essential oil, *CCI* chlorophyll content index, *LWC* leaf water content, *Carb* soluble carbohydrates, *Prol* proline, *SOD* superoxide dismutase, *CAT* catalase, *POX* peroxidase, *Mg* magnesium, *Cd* cadmium.

Principal Component Analysis (PCA) was utilized to comprehensively assess the relationships between cadmium toxicity, foliar application of magnesium, and various growth, physiological, and biochemical properties of mint plants. The PCA analysis effectively explained up to 87% of the variance observed in root and leaf characteristics, indicating a strong explanatory power of the chosen variables (Fig. 6). The PCA results revealed distinct patterns of association. Cadmium content, osmolytes, and antioxidant enzymes were positioned in the negative direction, suggesting an inverse relationship with plant growth parameters. This indicates that higher levels of cadmium toxicity are associated with increased osmolyte accumulation and heightened activity of antioxidant enzymes as a stress response mechanism. In contrast, magnesium content, CCI, root activity, and LA were aligned in the positive direction, signifying a positive correlation with improved plant growth and essential oil production. This positive association highlights the beneficial impact of magnesium foliar application on enhancing these physiological and growth parameters, thereby mitigating the adverse effects of cadmium toxicity. Furthermore, specific traits such as root growth and root activity demonstrated a strong and favorable correlation with magnesium content in plant tissues. This correlation underscores the critical role of magnesium in promoting root development and function, which are essential for overall plant health and productivity.

## Discussion

The findings of this study highlight that the foliar application of magnesium oxide particles, both in bulk and nano forms, contributes to enhanced plant resistance against cadmium stress. The significant improvement in plant growth and physiological performance under magnesium treatment can be attributed to its role in reducing oxidative stress, enhancing osmotic adjustment, and increasing magnesium content while reducing cadmium accumulation in plant tissues^12,17,24^. This leads to an increase in biological yield and essential oil production. In this research, the level of magnesium in the soil was found to be relatively low (Table 1). Under conditions of heavy metal toxicity, such as cadmium contamination, the absorption of many essential elements, including magnesium, is significantly reduced, even when these elements are present in sufficient quantities in the soil. Consequently, magnesium foliar spraying becomes necessary to ensure adequate magnesium uptake by the plants^27^. Cadmium stress stands out as one of the prevalent challenges faced by agricultural systems, triggering growth reduction in plants^38^. In line with this trend, the present research observed diminished plant growth under cadmium stress conditions, highlighting the adverse impact of this stressor. Our study observed a 32% reduction in plant biomass under cadmium stress, which aligns with previous findings in wheat, barley, and soybean^39–41^. This reduction is likely due to increased oxidative stress and decreased nutrient uptake, as indicated by the observed decrease in chlorophyll content and root activity in our study (Fig. 2).

The roots are the first part of the plant to face environmental stresses, such as exposure to heavy metals like cadmium. The decrease in root growth due to cadmium stress is linked to oxidative and osmotic stresses. PCA analysis also showed that root growth parameters have a negative correlation with cadmium content in plant tissues. This indicates that higher cadmium levels in the plant tissues are associated with reduced root growth, highlighting the detrimental impact of cadmium toxicity on the root system. Cadmium stress can hinder nutrient absorption and overall plant growth by reducing root activity. This metal can also inhibit the production of growth hormones such as auxins, which can lead to a decrease in root growth^11^. Applying magnesium through foliar spraying can help reduce cadmium accumulation in mint root tissues and improve root metabolism. This research suggests that magnesium can enhance root activity, leading to improved absorption of various nutrients. Additionally, magnesium can regulate the function of root ion channels, resulting in a reduction in the absorption of harmful environmental substances and an enhancement in the uptake of essential nutrients such as zinc^42,43^. The PCA results further demonstrate the positive effects of magnesium on root growth, highlighting its crucial role in promoting a healthy root system even under stress conditions.

The decline in plant biomass and leaf expansion under cadmium toxicity can be attributed to the increase in oxidative and osmotic stresses. The activation of plant defense mechanisms, such as the synthesis of osmotic regulators, incurs a high metabolic cost, which subsequently reduces the overall growth of the plant. In this research, PCA analysis reveals a negative correlation between osmotic regulators and parameters related to plant growth. This finding confirms that the plant's substantial physiological investment in creating resistance mechanisms comes at the expense of its growth. Optimal water levels are essential for cell growth and division within plant tissues^44^. In this research, a positive correlation was observed between plant water content and both leaf and root growth. This indicates that higher water content in the plant is associated with enhanced growth of these vital tissues, underscoring the importance of adequate hydration for optimal plant development. Cadmium stress negatively affects the root tissue and aquaporin functionality, leading to reduced water uptake by plants and subsequently hindering the growth and development of leaf cells^45,46^. The decrease in the chlorophyll content index of leaves due to cadmium toxicity may be associated with an increase in chlorophyllase enzyme activity and chlorophyll degradation^47^. Additionally, the generation of reactive oxygen species and lipid peroxidation can also contribute to the decline in chlorophyll levels within leaves^48^. Cadmium stress can prevent the absorption of nutrients, such as nitrogen and magnesium, in plant tissues. This can ultimately lead to a decrease in chlorophyll synthesis in plant leaves^49^. A decrease in plant growth, leaf expansion, and chlorophyll content under cadmium stress has been documented by various researchers^50,51^, further supporting the observed effects in this study.

The significant improvement in the growth and physiological performance of mint plants in response to magnesium treatments under cadmium stress can be attributed to several interconnected factors. Primarily, magnesium treatments appear to modulate the impact of oxidative stress, thereby contributing to improved osmotic adjustment and magnesium content, while simultaneously reducing cadmium accumulation within plant tissues. The increase in leaf area and chlorophyll content index observed after applying magnesium can be attributed to an increase in the concentration of this essential nutrient within the cells. Magnesium plays a pivotal role within plant cells, encompassing functions such as enzyme activation, participation in chlorophyll structure, synthesis of proteins and carbohydrates, and maintenance of ribosomal structure^12,52^. Magnesium is crucial in chlorophyll synthesis and activation of enzymes, directly impacting photosynthesis and plant growth^12^. The high correlation between the chlorophyll content in the leaves and the magnesium levels further underscores this relationship. By enhancing antioxidant enzyme activity (Fig. 3), magnesium helps mitigate oxidative stress caused by cadmium, thereby protecting cellular structures and functions. Additionally, magnesium's role in osmotic adjustment helps maintain cell turgor under stress conditions, as indicated by increased leaf water content and soluble carbohydrates (Fig. 4). The mitigation of harmful impacts caused by environmental stresses through magnesium foliar spraying is supported by previous research studies^53,54^. Additional studies have revealed the potential of nanomaterials in enhancing plants' resilience against environmental stressors. For instance, Zhou et al.^17^ demonstrated that nanomaterials can promote plant growth under heavy metal stress by facilitating the synthesis of stress-resistant hormones. Zou et al.^55^ highlighted that nano-nutrients have a significant ability to enhance antioxidant activity in plants, thereby reducing the harmful effects of cadmium stress.

The PCA results show a high and positive correlation between the activity of antioxidant enzymes and the cadmium content in plant tissues. This indicates that oxidative stress and the activation of antioxidant enzymes in response to it are among the most important defense mechanisms employed by mint plants under cadmium stress conditions. Cadmium accumulation in plant tissues often leads to oxidative stress, primarily caused by the incomplete transfer of electrons within photosynthetic systems^10^. Reactive oxygen species generated under these circumstances can cause structural damage to plant cells, particularly affecting components such as thylakoid membranes and the integrity of chlorophyll structure^1,56^. The association between cadmium accumulation and the increase in oxidative stress has been supported by other researchers^57,58^. Foliar spraying of magnesium, in both nano and bulk forms, emerges as a noteworthy strategy for reducing lipid peroxidation in mint leaves. This effect can be attributed to two well-established mechanisms: 1) Magnesium plays a central role in the structure of chlorophyll. Increased magnesium content within plant cells can enhance the efficiency of chlorophyll in facilitating electron transport. 2) Magnesium plays a crucial role in activating enzymes within plant cells, which, in turn, can enhance the activity of antioxidant enzymes within plant tissues^59,60^. The increase in antioxidant enzyme activity is a crucial mechanism by which plants counteract the harmful effects of ROS^61^. The researchers have identified a correlation between the activity of antioxidant enzymes and the magnesium content in plant tissues, emphasizing a dependable relationship in this context^62^.

Cadmium accumulation in plant tissues triggers osmotic stress, which leads to compromised root growth and damage to root aquaporins^63,64^. This stressor diminishes the availability of water for plant growth, a phenomenon substantiated by previous researchers^11^. In response to osmotic stress, plants increase the concentration of osmolytes, such as soluble carbohydrates, within their tissues. These osmoregulators facilitate increased water absorption by the plant. The foliar application of magnesium can increase the concentration of magnesium in plant tissues, thereby enhancing the synthesis of osmoregulators^65^. Magnesium plays a crucial role in carbohydrate synthesis, and an elevated internal concentration of magnesium can lead to an increased synthesis of soluble carbohydrates in plant cells^12^. Additionally, the foliar application of magnesium is observed to decrease the proline content in plant tissues. This outcome may result from an increase in chlorophyll synthesis within leaf tissues. Both proline and chlorophyll are derived from the precursor glutamate, and an increase in the synthesis of one of these compounds can result in a decrease in the quantity of the other within plant tissues^66^.

The reduction of cadmium accumulation in plant tissues following magnesium foliar spraying can be attributed to several mechanisms. One significant factor is the heightened activity of ion pumps in the vacuolar membranes, which enables the sequestration of heavy metals within vacuoles. This accumulation within vacuoles inhibits their transfer and subsequent accumulation in leaf tissues^67–69^. Furthermore, magnesium's ability to enhance the absorption of certain nutrients, such as zinc, by plant roots can contribute to limiting the uptake of cadmium. Elevated zinc concentration in plant tissues, achieved through mechanisms such as the inhibition of ion channels responsible for cadmium absorption, can lead to a reduction in cadmium content within plants^70^. It is noteworthy that in this study, the foliar application of nano-form magnesium was found to be significantly more effective in increasing the magnesium content in leaves compared to its bulk form. This distinction may be attributed to the unique physicochemical properties of nanoparticles, which enable plants to have a greater capacity for absorption due to their smaller size^71^. Despite the marginal change observed in the percentage of mint essential oil following magnesium foliar spraying, the increase in essential oil yield among plants treated with magnesium can be attributed to enhanced plant physiological performance. The increase in leaf area, chlorophyll content, and antioxidant enzyme activity contribute to stronger growth in plants exposed to cadmium stress. Ultimately, these improvements result in a higher yield of essential oil in mint plants. Despite the modest and statistically non-significant rise in plant weight attributed to magnesium foliar spraying, there was a noteworthy 17% increase in essential oil yield, a substantial enhancement from a statistical standpoint. This boost in essential oil production can be attributed to the amelioration of plant physiological conditions. Considering that cultivating medicinal plants for essential oil extraction presents a viable solution for utilizing heavy metal-contaminated lands, we strongly advocate for the adoption of magnesium foliar spray to enhance essential oil production in such plants. These results, while specific to mint (*Mentha crispa* L.), suggest that magnesium foliar spraying could be a viable strategy for enhancing heavy metal tolerance in other plant species and environmental conditions. While magnesium foliar spraying improved plant performance under cadmium stress, other factors such as soil nutrient levels and environmental conditions may also influence these results. Future studies should control for these variables to isolate the specific effects of magnesium.

## Conclusion

The results have revealed the adverse impact of cadmium stress on mint, resulting in decreased growth, impaired physiological performance, and reduced productivity. Conversely, the application of magnesium through foliar spraying under cadmium stress has been found to enhance plant productivity. This improvement is attributed to increased root growth, antioxidant activities, enhanced osmotic adjustment, and elevated chlorophyll synthesis within plant tissues. The foliar application of magnesium oxide nanoparticles increased plant biomass by 11%, leaf area by 24%, and chlorophyll content index by 10% under cadmium toxicity compared to control plants. Remarkably, although the nano form of magnesium resulted in a greater accumulation of magnesium in leaf tissues, it showed similar effects to the bulk form in terms of promoting plant growth and physiological efficiency. In general, foliar application of magnesium particles has excellent capacity for root growth and activity, and plant performance under toxic conditions. This study's limitations include the potential influence of uncontrolled environmental variables and soil nutrient levels, which could affect the generalizability of the findings. While our findings demonstrate the benefits of magnesium foliar spraying in mint, the long-term effects of magnesium foliar spraying on different plant species and its interaction with other nutrients under various environmental stresses are necessary to assess the sustained effects of magnesium supplementation on plant growth, yield, and quality. Comparing the effectiveness of magnesium supplementation through both soil and foliar applications could offer deeper insights into optimizing nutrient management strategies under heavy metal stress conditions. Future studies can focus on exploring repeated foliar applications of magnesium at different growth stages to determine the optimal frequency and timing for maximizing plant productivity. Incorporating these future research directions will provide a comprehensive understanding and better strategies to enhance the efficacy of magnesium in improving plant growth and mitigating heavy metal toxicity.

## Data Availability

The datasets used and/or analysed during the current study are available from the corresponding author on reasonable request.
